# Age as a primary driver of the gut microbial composition and function in wild harbor seals

**DOI:** 10.1038/s41598-022-18565-2

**Published:** 2022-08-27

**Authors:** A. Pacheco-Sandoval, A. Lago-Lestón, A. Abadía-Cardoso, E. Solana-Arellano, Y. Schramm

**Affiliations:** 1 Posgrado de Ciencias de la Vida, Centro de Investigación Científica y de Educación Superior de Ensenada, Ensenada, Baja California Mexico; 2 Departamento de Innovación Biomédica, Centro de Investigación Científica y de Educación Superior de Ensenada, Ensenada, Baja California Mexico; 3 Universidad Autónoma de Baja California, Facultad de Ciencias Marinas, Ensenada, Baja California Mexico; 4 Departamento de Ecología Marina, Centro de Investigación Científica y de Educación Superior de Ensenada, Ensenada, Baja California Mexico

**Keywords:** Microbial genetics, Ecological genetics

## Abstract

Dietary changes are the major variation cause in the composition of the gut microbiota. The short lactation phase in phocids provides an exceptional opportunity to explore the microbiota's response to a quick transition from a milk-based to a solid diet. We investigated the effects of age and sex on the gut microbiota of harbor seals in Mexico using rectal and fecal samples from pups and adults. 16S gene sequencing revealed age explains most of the observed variations in microbial composition. Individuals with frequent contact (pups—female adults) have major microbial similarities than those with little or no contact (pups—male adults). Overall, adults and females (regardless of sex and age, respectively) have a greater microbial richness; as seals grow, the core microbiome shrinks, and microbial diversity increases. We found pathways related to milk and chitin digestion in pups' microbiomes, indicating pups were transitioning to a solid diet. An enrichment of routes related to dramatic weight loss and body mass indicated higher metabolic stress in pups in late breeding season, when they are weaned and start intermittent fasting. Our findings highlight the host-microbiome interaction in harbor seals during late breeding season in response to food shifts and metabolic stress.

## Introduction

The relationship between age and microbiota composition is well established in humans. In newborns and babies, the gut microbiota is volatile and has a lower species richness than adults, becoming more stable and diverse during adulthood^1–6^. However, our understanding of how microbial communities developed across life stages in other species is limited, particularly in wild animals^7,8^.

In pinnipeds, previous research of the gut microbiota has focused mainly on adults^9–16^, with limited knowledge of pups' microbial community^7,17,18^. According to several studies, pups have lower microbial diversity than adults, and their microbial profiles vary as they age^7,18^.

The variations in the microbial structure between ages are primarily the result of diet-driven differences^14,19,20^. The transition from milk to a solid diet significantly impacts the gut microbiota composition and function, favoring microbial species that degrade dietary compounds^21,22^. Solid food consumption promotes bacterial richness and the evolution towards an adult-like microbiota^23,24^.

The distinct short lactation period in phocids offers a unique opportunity to study the microbial response to an earlier shift from a milk-based diet to fasting and solid food. Harbor seals (*Phoca vitulina*) are a remarkable example of maternal strategy and precocious offspring^20^. Females exhibit an "otariid-type" maternal strategy, fasting shortly after birth and undertaking foraging trips with their pups, who can swim within hours of birth^20,25–27^. Pups gain weight quickly and expend this energy during the post-weaning period^25^. Weaning marks the end of parental care and is a critical stage for offspring survival due to significant mass loss during the fasting phase^20,25,28^.

On the other hand, sex has been reported as a source of variation in the microbial composition of pinnipeds, especially in species with high sexual dimorphism^14,17,29^. Sex-related changes are most noticeable when females and males are grouped by age category, and these changes have been linked to different physiological capacities and prey consumption^14,17,29^. Even though harbor seals have a slight sexual dimorphism, metabolic stress differs between sexes and ages during the breeding season^30^. Females and pups are subjected to higher metabolic stress during the breeding and weaning periods due to weight fluctuations and fasting intervals^30^. In contrast, males experience lower metabolic stress, as indicated by cortisol stress hormone levels^30^. The gut microbiota is sensitive to metabolic stress, which alters the structure and functions of the micro-community^31^.

Changes in the harbor seal microbial composition may also occur between female and male adults with the pups since only females participate in parental care and stay near the pups during the breeding season^20,32^. Female adults and pups (both female and male pups) share more potential microbial sources as pups accompany their mother on foraging trips. Studies of cohabiting individuals demonstrated that direct and frequent contact leads to similitudes in the microbial composition due to a higher bacterial transmission, enhancing immune system development^33,34^.

Studying how microbial profiles and functions change throughout harbor seal's life will allow us to understand the role that microbes play during critical stress periods like lactation, weaning, and fasting. However, only the composition of the gut microbiota of adult harbor seals is known to date^12,13^; thus, more research is required to address this gap. This study presents the first report of microbial composition and functions in harbor seal pups. The aims of this research were: (1) compare the microbial composition among ages and sexes; (2) identify taxa responsible for differences in the microbial structure between adult and pups, as well as males and females; and (3) predict metabolic pathways that differ across ages and sexes in harbor seals.

## Results

### Comparison of fecal and rectal samples from pups

Fecal and rectal samples showed high variability between pup samples (see Supplementary Fig. S1 online); overall, Bacteroidota, Firmicutes, and Proteobacteria were the dominating phyla, while *Anaerobiospirillum*, *Bacteroides*, and *Alloprevotella* were the most abundant genera of amplicon sequence variants (ASVs). Of the 144 ASVs found, rectal and fecal samples shared 91, and the core microbiome included 31 members, accounting for 86.9% of the relative microbial abundance (see Supplementary Fig. S1 online). We found no differences between fecal and rectal samples in microbial richness (*p* = 0.959) or phylogenetic diversity (*p* = 0.716). Even though fecal samples had a greater microbial diversity than rectal samples (Shannon, *p* < 0.05), PCoA plots based on unweighted and weighted UniFrac distance matrices showed that regardless of sample type, samples from the same pup clustered together and remained within the pup group (see Supplementary Fig. S1 online).

### Composition of pup and adult microbiota

We sequenced the V4-16S rRNA region from rectal (pups = 12) and fecal (adults = 20) samples from 32 harbor seals from Natividad Island (see Supplementary Fig. S2 online). Pups' rectal samples contained mucosa and also rectal content due to the internal rubbing technique used during the sampling. Sex assignment was successful in 90% of the adult samples, resulting in a final sex proportion of seven males and five female pups and 11 females, seven males and two adults with unassigned sex. For the comparative analyses, only sex-assigned samples were used.

**Figure 1:**
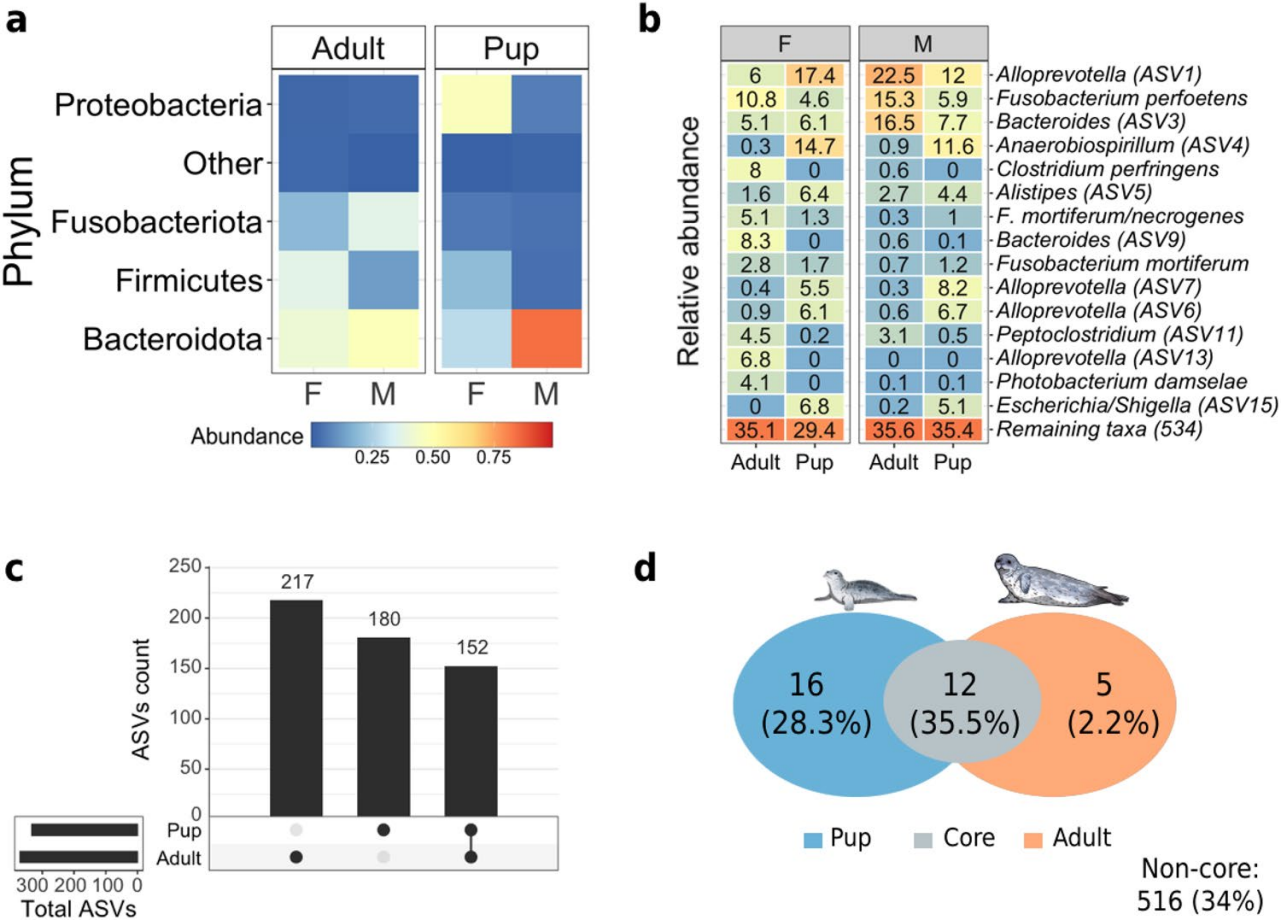
Microbiome composition of harbor seal pups and adults. (**A**) Phyla with a relative abundance > 1%. (**B**) Top 15 most abundant ASVs detected in the microbiome of harbor seals. The number of ASVs is shown when the species is unassigned. (**C**) Number of unique (single dot), shared (connected dots), and total (horizontal bars) ASVs among ages and sexes. (**D**) The core microbiome of harbor seals among pup and adult. The core group consisted of ASVs with an 80% prevalence and a relative abundance ≥ 0.01%. The Venn diagram shows the number of ASVs and their relative abundance in each core group.

After the filtering process, we retained 1,749,475 read pairs clustered into 572 ASVs (see Supplementary Table S1 online) represented by two Kingdoms (Archaea and Bacteria), 16 phyla (see Supplementary Fig. S3 online), 27 classes, 69 orders, 98 families, and 128 genera. The dominant phyla were: Bacteroidota, Firmicutes, Fusobacteriota, and Proteobacteria (Fig. 1a), and only the phyla Bacteroidota and Proteobacteria were more abundant in pups than in adults. The most frequent bacteria ASVs in adults were *Alloprevotella*, *Fusobacterium perfoetens,* and *Bacteroides*, and the pup microbiome was dominated by *Alloprevotella*, *Anaerobiospirillum*, *Escherichia/Shigella* ASVs (Fig. 1b). Microbial profiles revealed greater intraspecific variation between individuals of the same age (see Supplementary Fig. S4 online), but in general, total and unique ASV counts were higher in adults than pups, with both ages sharing 152 ASVs (Fig. 1c).

According to alpha diversity estimates, the harbor seal microbiota richness differed between ages but not sexes. Comparison between females and males was not significantly different for phylogenetic diversity (*p* = 0.430) or richness (*p* = 0.778). Only the Shannon index indicated sex differences, with female harbor seals having a greater mean diversity value than males (Shannon, *p* < 0.001). Even though pups had higher mean values for richness (*p* = 0.046) and phylogenetic diversity than adults (*p* = 0.002), their microbiota was dominated by fewer microbial members. In contrast, adults hosted more diverse bacterial communities (Shannon, *p* < 0.001).

The core microbiota of pups and adults had a total relative abundance of 35.5% (Fig. 1d) and consisted of 12 members represented by six genera and unknown members of the families Fusobacteriaceae and Erysipelotrichaceae (see Supplementary Table S2 online). *Fusobacterium perfoetens* was the most abundant species in this core (9.7%). Pups' core microbiota had more microbial members than the core group of adults, with 16 distinct members accounting for 28.3% of the relative abundance and *Alloprevotella* dominating the pups' core (14.3%). In contrast, the core microbiota of adults consisted of five bacterial members (Fig. 1d), representing 2.2% of the relative abundance, with *Colidextribacter* the most abundant bacteria (see Supplementary Table S2 online).

### The effect of age and sex on the microbial structure of harbor seals

**Figure 2:**
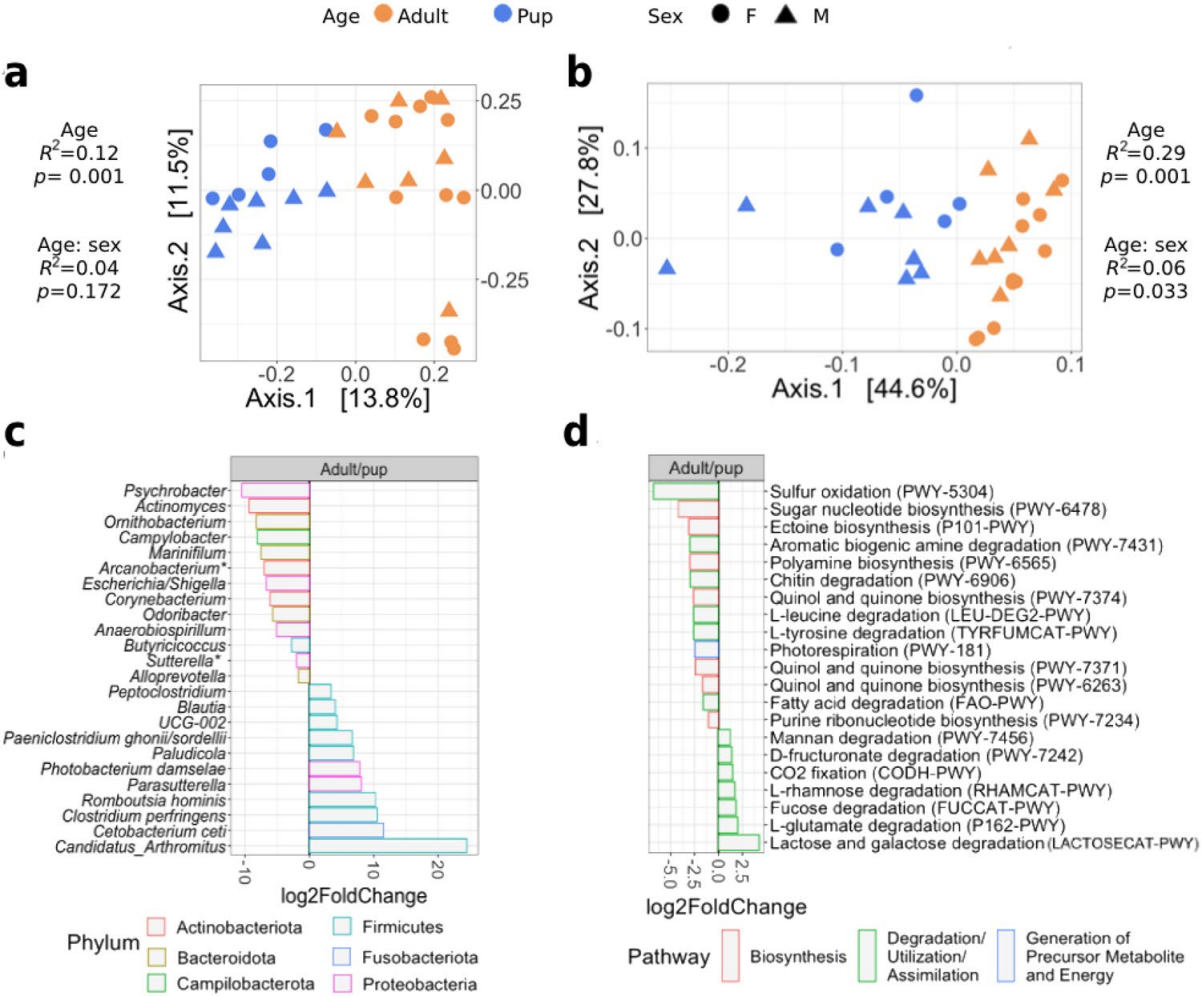
Age explains the major differences in microbial diversity of harbor seals. Principal Coordinate Analysis (PCoA) on unweighted (**A**) and weighted (**B**) UniFrac distances based on age and sex groups. Differential abundance analysis reveals changes between adults and pups in taxa (**C**) and EC pathways (**D**). Genus and pathways with a log_2_-fold-change > 0 are significantly more abundant in adults. *Multiple species assignments.

The overall comparison of age and sex revealed that age explained most of the variation in the microbial community composition of harbor seals. Age influenced the composition (R^2^ = 0.12, *p* = 0.001) and abundance (R^2^ = 0.29, *p* = 0.001) of microorganisms, as evidenced by PCoA results based on UniFrac distances (Fig. 2a,b). Sex did not affect the microbial composition (R^2^ = 0.04, *p* = 0.172), but when the abundance of the microorganism was evaluated (weighted UniFrac), the interaction between age and sex revealed significant differences (Fig. 2b). In contrast, we did not find significant differences between sex categories, between female and male pups, and between female and male adults (see Supplementary Table S3 and Fig. S5 online).

DESeq2 results revealed 24 genera responsible for differences in the microbial composition between age groups (Fig. 2c) and 21 EC pathways (Fig. 2d) that differed between adults and pups. The following taxa were identified as members of the pup core microbiota and were more abundant in pups than in adults: *Actinomyces*, *Escherichia/Shigella*, *Odoribacter*, *Anaerobiospirillum*, *Butyricicoccus*, *Sutterella,* and *Alloprevotella*. The genera *Blautia* and *Parasutterella* were more prevalent in adults than in pups and were part of the adults' core microbiota.

We discovered that most changes in microbiota functions between ages are associated with the group of Degradation/Utilization/Assimilation pathways. Of the metabolic pathways of interest, quinol and quinone biosynthesis, chitin, fatty acid, and amino acid degradation were significantly higher in pups. In contrast, adults showed a higher abundance of enzymes related to sugar degradation (Fig. 2d). All functional inferences in this study are based on PICRUSt2 analysis of 16S rRNA gene sequences.

### Differences in the microbiota composition between female and male adults with pups

PERMANOVA pairwise test comparison in the age and sex interaction revealed differences in the microbial composition between all the comparative cases (see Supplementary Table S3 online). However, we decided to compare the microbial composition of individuals in close contact (female adults vs. pups) to those with little or no contact (male adults vs. pups), as we expected that individuals in close contact might had fewer differences in their microbial profiles.

**Figure 3:**
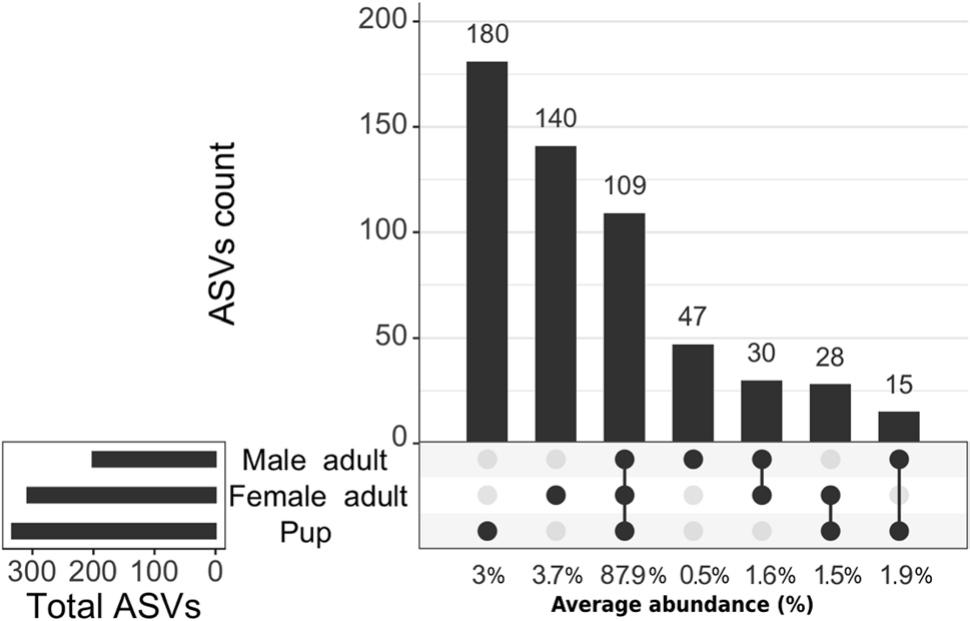
Differential composition between female/male adults and pups. The upset diagram shows the number of unique (single dot), shared (connected dots), and total ASVs (horizontal bars) among adults and pups. Percentages represent the average abundance of each group.

Pups had more exclusive ASVs, and they shared more ASVs with female adults (Fig. 3). In general, shared ASVs had a mean relative abundance < 0.5% (see Supplementary Table S4). In the core group of female adults and pups, ASVs from the genus *Bacteroides* and unknown bacteria from the families Ruminococcaceae and Oscillospiraceae were more common. This core represented 1.6% of the average abundance of the harbor seal microbiome. In contrast, male adults had fewer unique ASVs than females and shared only 15 ASVs with pups. Overall, adults had more ASVs than pups, and both ages shared 109 ASVs, representing 87.9% of average microbial abundance in the harbor seal samples (Fig. 3).

### Microbial differences between female adults and pups

**Figure 4:**
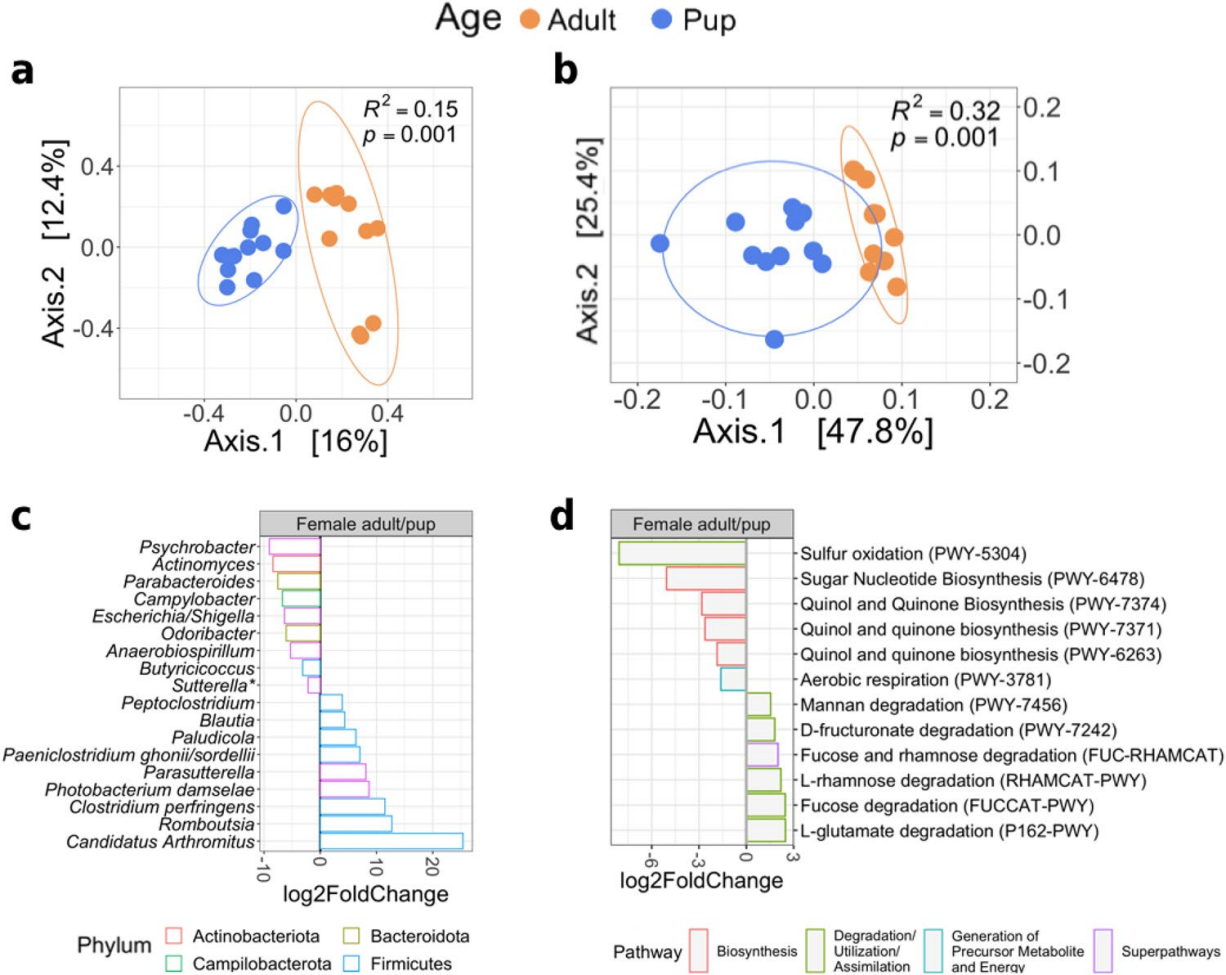
Changes in microbial diversity of female adults and pups harbor seals. Principal Coordinate Analysis (PCoA) on unweighted (**A**) and weighted (**B**) UniFrac distances based on females; ellipses represent a 90% confidence interval. Differential abundance analysis reveals changes between female adults and pups in taxa (**C**) and EC pathways (**D**). Genus and pathways with a log_2_-fold-change > 0 are significantly more abundant in female adults. *Multiple species assignments.

The microbial community's composition (Fig. 4a) and abundance (Fig. 4b) differed between female adults and pups. Individual dispersion within groups was homogeneous, implying that ordinations were reliable. When we only evaluated the presence/absence of microorganisms, better separation between groups was achieved (unweighted UniFrac; Fig. 4a), but when we included the abundance of microorganisms (weighted UniFrac; Fig. 4b), a greater fraction of the variation was explained by the groups (*R*^2^ = 0.32, *p* = 0.001).

In the microbiome of pups and adult females, we discovered 18 taxa with varying abundance (Fig. 4c). The main changes in the microbiota composition between females and pups are related to the Firmicutes phylum, which was highly abundant in female adults; from this phylum, only the genus *Butyricicoccus* had a greater abundance in pups.

Pup and adult females had 12 EC pathways with differential abundances. Female adults had considerably higher levels of pathways involved in the degradation of complex carbohydrates (mannan), monosaccharides (fucose and rhamnose), sugar acid (D-fructuronate), and amino acids (L-glutamate) (Fig. 4d).

### Microbial differences between male adults and pups

**Figure 5:**
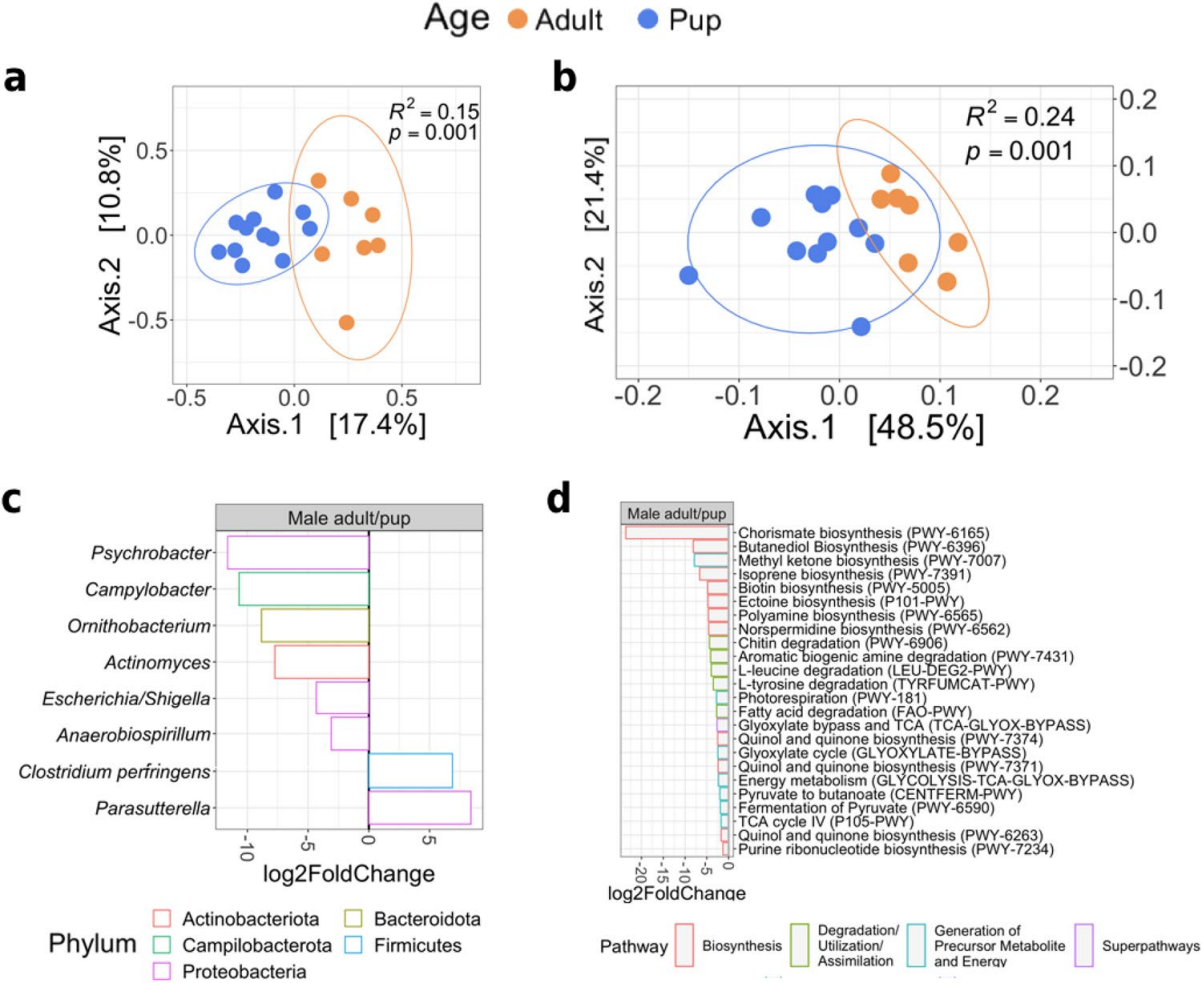
Changes in microbial diversity of male adults and pups harbor seals. Principal Coordinate Analysis (PCoA) on unweighted (**A**) and weighted (**B**) UniFrac distances based on males; ellipses represent a 90% confidence interval. Differential abundance analysis reveals changes between male adults and pups in taxa (**C**) and EC pathways (**D**). Genus and pathways with a log_2_-fold-change > 0 are significantly more abundant in male adults.

The comparison between male adults and pups revealed differences in the microbial community's composition (Fig. 5a) and abundance (Fig. 5b). The microbial dispersion between individuals did not vary within groups when considering the composition (betadisper-test, *p* = 0.900) and microorganisms' abundance (betadisper-test, *p* = 0.257). When we considered the abundance, a greater proportion of the variability between groups was explained (*R*^2^ = 0.24, *p* = 0.001).

We observed some changes in the microbiota composition between groups, detecting eight genera with different abundances (Fig. 5c). Male adults had a higher prevalence of *Clostridium perfringens* and *Parasutterella* than pups. As demonstrated in comparing female adults and pups, pups had a higher abundance of the genera *Psychrobacter, Campylobacter, Anaerobiospirillum, Actinomyces,* and *Escherichia/Shigella*, with the last two being members of the core microbiome in pups.

A total of 24 functional pathways differed between males, all of which were more abundant in pups (Fig. 5d). Most of the observed changes in the microbiome function were related to biosynthesis routes. Degradation pathways were represented by amino acids (l-leucine and l-tyrosine), fatty acids, natural polysaccharides (chitin), and aromatic compounds (amine). Regardless of adult sex, the enzyme pathways related to quinol and quinone biosynthesis (PWY-7374 and PWY-7371) were more prevalent in pups.

## Discussion

We studied the influence of age and sex on the composition and function of the gut microbiota of harbor seals using a 16S rRNA amplicon sequencing strategy. We discovered that age explains most of the observed variations in the microbial composition. Sex-related differences between adults and pups were more noticeable when the abundance of microorganisms was considered. Female adults have more total and unique ASVs and share more ASVs with pups than male adults. Overall, female harbor seals have a greater diversity of microorganisms than males. As seals grow, the core microbiome shrinks, and microbial diversity increases.

We found similarities in the community structure of pups' fecal and rectal samples, and both samples clustered together in PCoA plots (see Supplementary Fig. S2 online). The high number of ASVs shared between fecal and rectal samples suggests that both types of samples are representative of one another and may be useful for providing insight into the bacterial community present in the large intestines of harbor seals^14^. Rectal samples are most useful and reliable for characterizing the gut microbiota when the feces are liquid and more likely to be contaminated with bacteria from the environment, such as harbor seal pup feces.

We found that age is a key factor that influences the composition and function (based on PICRUSt2 analysis of 16S rRNA gene sequences) of the gut microbiota of harbor seals. Previous research in other pinnipeds has also identified age as a significant driver of gut microbiota composition^7,14,18^. Changes in microbial structure between ages might result from diet-driven differences. Harbor seal pups feed mainly on milk and are weaned after four to six weeks^20,28,35^, while the adult diet is based primarily on benthic or demersal fishes and cephalopods^13,36–38^.

Pup-enriched metabolic pathways indicate that pups were transitioning from nursing to a solid diet. Pinniped milk is rich in fat (60%), and protein concentration (10%)^39^ and the most abundant amino acids are glutamate, proline, and leucine^39^. The leucine degradation pathway was more abundant in pups than in adults, and glutamate was more abundant when pups were compared to adult males. Enrichment of routes related to the degradation of fatty acids and some amino acids abundant in milk could indicate that pups were still nursing at the sampling time.

However, the increase of chitin degradation, a characteristic compound of the invertebrate exoskeleton, is consistent with pups feeding at a lower trophic level than adults^37^. Some studies reported that initially newly-weaned harbor seals feed on small crustaceans and fishes^28,36^. Crustaceans play a major role in the pups diet, suggesting that pups are inefficient in capturing fish at this age^28^. As their foraging and diving skills improve, pups switch to an adult diet, but ingestion of solid food may occur while the pup is still nursing^40^.

Harbor seal breeding season has not been described on Natividad Island; however, available information exists for the colony on San Roque Island, close to our research area. Fernández-Martín^41^ studied the 2014 San Roque Island breeding season and found that it lasted from the end of December to the end of March, with the peak of pups occurring on February 7th and the lactation period lasting 21 days. The long breeding season (13.43 weeks), in comparison to other northern colonies, showed that pupping was less synchronized. If harbor seals from Natividad Island have a similar pupping period than San Roque Island, then our sampling was near the end of the breeding season, which is consistent with our finding of the transition from milk to solid food.

Weaning transition studies have found an increased abundance of facultative anaerobic bacteria, such as Escherichia/Shigella^42^, characteristic of harbor seal pup microbiota. Also, the higher number of core microbiome members shared between post-weaning northern elephant seals (*Mirounga angustirostris*)^17^ and our pup samples support the idea that harbor seal pups were at the beginning of the post-weaning period.

We identified 12 core microorganisms that persisted from young age to adulthood (Fig. 1d). The maintenance of these microbial species through life suggests they may provide functions unrelated to diet, as diet varies greatly between ages. Instead, this core microbiome may be involved in developing the immune system, and its preservation is ensured through vertical transmission^14^. Overall, we found that adults have fewer members in their core microbiome than pups, which has been observed in other seal species^18^.

The existing research on the pinniped microbiome agrees that the genera *Bacteroides* and *Fusobacterium* and unidentified members of the families Fusobacteriaceae and Lachnospiraceae are part of the core microbiome in this Carnivora clade^9,13,14,17,18^. This suggests that the mentioned genera play an essential role in pinniped host evolution, probably related to immune functions.

Available studies of adult harbor seal microbiota have also identified the genera *Bacteroides*, *Fusobacterium, Anaerobiospirillum* as members of the core microbiome^12,13^. Some of the bacteria detected in the core microbiome of adult seals, such as *F. mortiferum*, *F. perfoetens*, *Colidextribacter*, and an unidentified Oscillospiraceae, have also been reported in sourthern elephant seals (*Mirounga leonina*) and Weddell seals (*Leptonychotes weddellii*) adults microbiomes^29^.

*Fusobacterium*, *Bacteroides, Odoribacter, Anaerobiospirillum, Escherichia/Shigella, Phascolarctobacterium,* and *Collinsella* are part of the pup microbial core and have also been identified as pup microbiome members in post-weaning northern elephant seals living on San Benito Island^17^, which is near to our sampling site (see Supplementary Fig. S1 online).

In comparison to other phocid species, harbor seal pups are particularly precocial at entering the sea shortly after birth and swimming with their mother throughout the lactation period^20,26,27^. Swimming requires higher energy expenditure, so the post-weaning fast is shorter than other species due to less energy storage during lactation^28^. Harbor seal pups lost 21% of weaning mass over the first 5 weeks of the post-weaning stage and fasted an average of 15–17 days. Body fat comprises 32.8% of body mass at the end of weaning but declines to 12% after 26 days post-weaning^28^. Also, protein decrease from 5.5% at birth to 2.5% near weaning^28^.

Dramatic weight loss and body mass have been related to changes in the gut microbiota composition in different species^17,42–44^. A recent study of patients with cachexia, a body condition characterized by weight loss, muscle wasting, and fat changes, revealed a significant reduction of catabolic pathways related to complex carbohydrates (mannan), sugar derivatives (glucuronide, fructuronate), fatty acid degradation, and amino acid (l-glutamate, l-leucine and l-tyrosine) compared to the non-cachexia group^44^. These pathways were significantly decreased in pups compared to adults, suggesting that metabolic stress in female adults is lower than in pups during the breeding season^30^.

Although females are susceptible to metabolic stress and weight loss during the breeding season, our findings suggest that pups are more prone to metabolic stress and that the post-weaning phase is crucial for their survival. The small size of female harbor seals impedes prolonged fasting and nursing due to fewer fat deposits; hence females feed during the nursing period to restore lost fat^25,32^. However, harbor seal pups grow slowly and are leaner at weaning than other species that do not swim before weaning^25^.

Several quinol and quinone biosynthesis pathways were enriched in pup microbiota; these components mediate respiratory electron transport playing a key role in the energy generation process^45^. Quinones are also growth factors for bacteria, and among the genera identified as quinone producers were *Sutterella*, *Escherichia coli,* and *Bacteroides*^46^*,* which were abundant in pup microbiota and are also members of their core. As an individual grows, the diversity of microbes increases^47^; thus, the role of these bacteria in early life is fundamental in promoting the growth and diversification of other microbial species.

Sex has minimal effect on the microbial community of harbor seals as Shannon index differences were the only evidence of sex-related variations in the harbor seal microbiome, with females having a higher microbial diversity than males. Female adults modify their aquatic distribution, fasting duration, and foraging activity during the breeding and pupping season^32^. These changes vary according to females' body size and the presence of pups, with bigger females able to fast for more extended periods than smaller ones^48^. Also, females with no pups did not restrict their foraging range^32^. In contrast, when females start making foraging trips, males limit their distribution range and concentrate near feeding locations or transit routes to maximize contact with oestrus females^49^. These female behavioral changes may be responsible for the greater variety and diversity observed in the microbial composition of female adults, as shown by the higher number of ASVs detected in females than in males (Fig. 3).

When comparing the beta diversity between sexes, no changes in the microbiota composition between females and males were observed (regardless of age), as seen in other phocids with low sexual dimorphism, like the leopard seal^14^. Changes in the microbiota composition between females and males have been reported in pinniped species with high sexual dimorphism, like northern elephant seals, where variations in the gut microbial structure have been linked to differences in dispersion capability and foraging strategies between sexes^14^.

Adult harbor seals have lower sexual dimorphism than other phocids, with females typically smaller than males^20^. In this species, differences in the diet, range distribution, and foraging area are mainly related to the body size and mass of the specimen rather than sex^48,50,51^. The presence of considerable intrasexual variation in diet and foraging range indicates that the animal's physiological capabilities determine differences in both factors rather than sex^48,50^. Therefore, as in other phocids with low sexual dimorphism, sex may not be a major factor in the diversity of the gut microbial community^14,29^.

Also, we did not observe differences in the microbial composition between the pups' sexes. Some studies have reported that mean birth mass, mass loss rate, and fasting duration in the post-weaning period did not differ between female and male pups, indicating that physiological and metabolic capabilities are similar among sex categories^28,52^.

We found similarities in the community structure of female adults and pups, as both shared more ASVs than male adults did with pups. The members of the shared micro-community were relatively low in abundance and phylogenetically diverse, and they formed part of the "rare biosphere" in the harbor seal gut. As in humans, many studies suggest that rare taxa are important components of the gut and could contribute either independently or collectively to various metabolic functions crucial to seal health^53^.

Female adults and pups often interact and share more potential microbial sources, resulting in a greater number of shared ASVs. During the breeding and pupping season, females and pups tend to form smaller and isolated groups in areas preferred for parturition and nursing^54^, and pups accompany their mother during foraging trips until weaning^20,32^. Male harbor seals have little or no interaction with pups since they do not participate in parental care and only restrict their distribution range during the mating season^49^. Studies of cohabiting individuals demonstrated that direct and regular contact leads to similarities in the microbial composition due to an increased bacterial transmission, which enhances immune system development^33,34^.

Within the group of microbes with differential abundances between ages, we detected some potential pathogens, such as *Actinomyces*, *Corynebacterium,* and *Escherichia/Shigella*, with the last two reported in pups^55,56^. Adults have a higher abundance of *Photobacterium damselae,* and *Cetobacterium ceti* and pups have a higher abundance of *Psychrobacter* and *Campylobacter*, all of which have been associated with seals foraging near salmon farms^57^. On Natividad Island, a cooperative fishing society, which focuses mainly on abalone farming and lobster fishing, represents the main source of economic income for the residents.

*P. damselae* is a pathogen of diverse species, including fish, crustaceans, and cetaceans, causing ulcerative lesions and hemorrhagic septicemia^58^. This species affects farmed fishes' species and is considered a threat to mariculture and aquaculture farms because disease produced by this bacterium causes mortalities^59^, resulting in financial losses. Humans are also vulnerable to this species, producing severe wound infections and necrotizing fasciitis^60^. This species may be of concern to harbor seals and humans living nearby, as pathogen transmission from the water to humans can occur^59^. Further research is necessary to determine whether these microorganisms are pathogenic or commensal species of harbor seals and address the potential transmission of these bacteria from the environment to humans.

## Methods

### Study site and sample collection

Samples were collected in Natividad Island, located 9.3 km off the West Coast of Baja California, Mexico (see Supplementary Fig. S1 online). This island is part of the Vizcaino Biosphere Reserve—a protected natural area—and is inhabited by one of the most abundant harbor seal colonies in Mexico, with the slightest variance in population abundance throughout the year^61,62^.

During the 2020 breeding season (March), we collected the inner parts of 20 fresh feces samples from juvenile/adult harbor seals and obtained microbial samples from 12 hand-captured pups by rubbing a sterile Flock swab® into the rectum (samples contained both rectal mucosa and content). We captured the pups with a light cotton net tied to a flexible plastic ring and held them for the time necessary to take the samples, which lasted less than 10 min. Also, when capturing pups, we had the opportunity to collect fresh fecal samples from two pups. Both types of samples were maintained at − 80 °C until further analysis in a sterile tube containing RNA later® (Sigma-Aldrich). The sex of the pups was determined by visual examination.

All samplings and fieldwork procedures were conducted under the Mexican Ministry for Environment and Natural Resources' permit SGPA/OGVS/11448/19.

### Ethic statement

To capture the pups, we selected those on land, far from the shoreline, to catch them before they got to the water during the approach. We approached carefully (crawling, hiding behind rocks) to avoid being detected and minimize disturbance to the colony. During capture, no additional procedures or clinical analyses were performed. However, in all cases, captured pups were in good physical condition and showed no apparent injury or disease. The collection of feces and rectal samples was approved by the Research and Postgraduate Ethics Committee of the Autonomous University of Baja California. All sampling and animal handling were performed strictly according to the approved guidelines and regulations.

### DNA extraction, amplification, and sequencing

We extracted bacterial DNA from approximately 250 mg of fecal material from fecal and rectal samples using the QIAamp Fast DNA Stool Mini Kit (QIAGEN). Before DNA extraction, we added 1 mL of sterile PBS to the pup swabs, briefly vortex-mixed the tubes, and removed the swab following a 15 min centrifugation at 10 000×*g* at 4 °C. For both samples, we performed bacterial lysis at 95 °C following the manufacturer's protocol. DNA quality was visualized by agarose gel electrophoresis, and DNA concentration was quantified using the Qubit dsDNA HS kit (High Sensitivity) on a Qubit 3.0 fluorometer (Life Technologies).

We amplified the hypervariable IV region of the 16S rRNA gene using the 515F-806R primers^63^ with a dual-index strategy^64^ for paired-end sequencing. PCR reactions were set up as triplicates for each sample in 25 µl reactions, and PCR program consisted of 95 °C for 5 min followed by 30 cycles with 98 °C for 9 s, 55 °C for 60 s and 72 °C for 90 s and a final extension step at 72 °C for 10 min. PCR products were visualized on an agarose gel, pooled, and quantified with a Qubit dsDNA BR Assay kit (Thermo Scientific) on a Qubit 3.0 fluorometer (Thermo Fisher Scientific). We normalized PCR products with the SequalPrep Normalization Plate (96) kit (Applied Biosystems™) following the manufacturer's protocol ﻿ (final concentration: 1–2 ng/μl).

Sequencing was performed at Metagenomics Lab of the Center for Scientific Research and Higher Education at Ensenada (CICESE) on an Illumina MiSeq platform using the MiSeq Reagent Kit v2 (300 cycles) as described by Kozich et al.^64^.

### Genetic sexing

We modified the Robertson et al.^65^ protocol to determine the sex of harbor seals from fecal samples. This method allows molecular sex identification using two primer sets that amplify small regions of the *SRY* and *ZFX/ZFY* loci. Instead of using real-time PCR and the High-Resolution Melting (HRM) technique to compare the melting curves of the DNA strands (original method), the amplifications were done using multiplex PCR. Amplification was performed in a 25 µL reaction with a final concentration of 5 × MyTaq™ reaction buffer (Bioline), 0.1 U MyTaq™ DNA Polymerase (Bioline), 400 nM each primer, and 5–15 ng of DNA. The PCR program consisted of 95 °C for 15 min followed by 45 cycles of 95 °C for 30 s, 60 °C for 30 s and 72 °C for 30 s. PCR products were visualized on a 2% agarose gel (115 V for 75 min). A band of approximately 168 bp belonging to the ZFX/ZFY gene is expected in females, while two bands of 224 bp and 168 bp corresponding to the SRY and ZFX/ZFY genes, respectively, are expected in males. All amplifications included: (a) triplicates of each sample, (b) positive controls corresponding to harbor seal pup samples of known sex (one female and one male), and (c) negative controls.

### Sequence analysis

In the paired end-mode, forward and reverse primers were trimmed with Cutadapt v2.8^66^. The sequencing reads were filtered, denoised, merged, and assessed for chimeras following the DADA2^67^ pipeline (https://benjjneb.github.io/dada2/tutorial.html) on the R environment with default parameters unless specified. We used the *pool* = "pseudo" option, resulting in 692 unique amplicon sequence variants (ASVs).

Using DADA2's native RDP Bayesian classifier, taxonomy was assigned against the Silva 138 database with a *minBoot* = 80. We used the *assignSpecies* option for species assignment, allowing for multiple assignments (*returnMultiple* = TRUE). We discarded singletons, *phyla* with one read, unclassified Kingdom, and mitochondria/chloroplast assignments for the following analysis.

### Composition of harbor seals microbiota

We defined the core microbiota of pups and adults as the ASVs present in at least 80% of the samples with a relative abundance ≥ 0.01%. We only considered the samples with assigned sex for the analysis. Shared and unique ASVs between groups were visualized using UpsetR package v.1.4.0. These and further analyses and plots were performed in RStudio v.3.6.2^68^ using *phyloseq* v.1.30^69^, *vegan* v.2.5.7^70^, *ampvis2* v.2.6.4^71^, and *ggplot2* v.3.3.5^72^ packages, primarily.

### Alpha diversity

Alpha diversity metrics were estimated with the raw counts of ASVs and included: richness (number of ASVs), Shannon, and Faith's phylogenetic diversity (PD). To investigate whether alpha diversities differ between groups, we fitted a linear mixed model with alpha diversity as the response variable and age (pup/adult) and sex (male/female) as fixed effects. We performed an analysis of variance (ANOVA) to determine the significance of the predictor variables and to test the differences between groups. A Shapiro–Wilk test was used to confirm normality and Bartlett's test to determine homoscedasticity. The Shannon diversity indexes were compared using Hutcheson's t-test available in the R library *ecolTest*^73^.

### Beta diversity

We used the *DESeq2*^74^ package to transform ASV counts (ASV counts < 4 were removed) into a DESeq2 object with the model: ~ sex + age, and the transformed data were used in the following analysis. We applied two major approaches to evaluate changes in the microbial composition among samples; the first involved a broad comparison between groups (age and sex), and the second focused on evaluating the interaction of the variables (age*sex).

We calculated weighted and unweighted UniFrac distance matrices^75,76^ using *phyloseq*^69^ and visualized them through Principal Coordinate Analysis (PCoA) with 90% confidence intervals computed using the *stat_ellipse* function. The effect of age, sex, and the interaction of these variables were assessed using a permutational analysis of variance (PERMANOVA; *adonis* function) with UniFrac distances matrix^76^ and 999 permutations in the R *vegan*^70^ package. We performed pairwise comparisons between age and sex and corrected for multiple comparisons using the *pairwise.adonis* function^77^.

### Differential abundance analysis

We collapsed ASVs at the genus level with *phyloseq*^69^ and used DeSeq method to test for differential abundances of genera between age and sex groups, between female adults -pups and between male adults- pups. To identify genera with consistent differential abundance among the groups, adjusted *p*-value < 0.01 was considered, and we only evaluated the comparative groups that showed statistically significant differences in their beta diversity. We compared ASVs at the genus level, and when available, we added the species classification.

### Functional analysis PICRUSt2

We processed samples in the PICRUSt2 software^78^ to predict the metagenomic functional composition of the harbor seal microbiota based on ASV abundance. Raw ASV abundance table with prevalence > 1 was imported into the Python programming environment v.3.7.6, and the PICRUSt2 pipeline was used with default parameters (https://github.com/picrust/picrust2/wiki/PICRUSt2-Tutorial-(v2.4.1). The Enzyme Classification (EC) and MetaCyc^79^ databases were used as references for pathway annotation to predict the microbial gene content and the output fed the downstream analysis, including differential test abundance with DESeq2.

DESeq2 was also used to transform EC counts from PICRUSt2 using the model: ~ sex + age (also used in ASV abundances); ECs present in only one sample and with a count < 30 were removed. An adjusted p-value < 0.01 and a log twofold change greater than ± 1 were used to identify ECs with consistent differential abundance among groups. We evaluated only comparative groups that showed statistically significant differences in their beta diversity.

## Supplementary Information

Supplementary Information.

Supplementary Table S1.

Supplementary Table S2.

Supplementary Table S4.

## Data Availability

Raw 16S rRNA gene sequence fastq files are deposited in the SRA database of the NCBI (BioProject PRJNA803311); metadata are also stored in the SRA (BioProject PRJNA803311). All data analyzed during this study are included in the published article and its supplementary information files.

## References

[1] Koenig JE (2011). Succession of microbial consortia in the developing infant gut microbiome. Proc. Natl. Acad. Sci..

[2] Bäckhed F (2015). Dynamics and stabilization of the human gut microbiome during the first year of life. Cell Host Microbe.

[3] Tanaka M, Nakayama J (2017). Development of the gut microbiota in infancy and its impact on health in later life. Allergol. Int..

[4] Xu C, Zhu H, Qiu P (2019). Aging progression of human gut microbiota. BMC Microbiol..

[5] Yatsunenko T (2012). Human gut microbiome viewed across age and geography. Nature.

[6] Nagpal R (2017). Ontogenesis of the gut microbiota composition in healthy, full-term, vaginally born and breast-fed infants over the first 3 years of life: A quantitative bird’s-eye view. Front. Microbiol..

[7] Smith SC, Chalker A, Dewar ML, Arnould JPY (2013). Age-related differences revealed in Australian fur seal *Arctocephalus pusillus doriferus* gut microbiota. FEMS Microbiol. Ecol..

[8] Janiak, M. C. *et al.* Age and sex-associated variation in the multi-site microbiome of an entire social group of free-ranging rhesus macaques. *Microbiome* **9**, (2021). 10.1186/s40168-021-01009-w PMC7986251 33752735

[9] Toro-Valdivieso C, Toro F, Stubbs S, Castro-Nallar E, Blacklaws B (2021). Patterns of the fecal microbiota in the Juan Fernández fur seal (*Arctocephalus philippii*). MicrobiologyOpen.

[10] Medeiros AW (2016). Characterization of the faecal bacterial community of wild young South American (*Arctocephalus australis*) and Subantarctic fur seals (*Arctocephalus tropicalis*). FEMS Microbiol. Ecol..

[11] Bik EM (2016). Marine mammals harbor unique microbiotas shaped by and yet distinct from the sea. Nat. Commun..

[12] Numberger D, Herlemann DPR, Jürgens K, Dehnhardt G, Schulz-Vogt H (2016). Comparative analysis of the fecal bacterial community of five harbor seals (*Phoca vitulina*). MicrobiologyOpen.

[13] Pacheco-Sandoval, A. *et al.* The Pacific harbor seal gut microbiota in Mexico: Its relationship with diet and functional inferences. *PlosOne* **14**, (2019). 10.1371/journal.pone.0221770 PMC6715212 31465508

[14] Nelson TM, Rogers TL, Carlini AR, Brown MV (2013). Diet and phylogeny shape the gut microbiota of Antarctic seals: A comparison of wild and captive animals. Environ. Microbiol..

[15] Glad T (2010). Ecological characterisation of the colonic microbiota in Arctic and sub-Arctic seals. Microbiol. Ecol..

[16] Delport TC, Power ML, Harcourt RG, Webster KN, Tetu SG (2016). Colony location and captivity influence the gut microbial community composition of the Australian sea lion (*Neophoca cinerea*). Appl. Environ. Microbiol..

[17] Stoffel MA (2020). Early sexual dimorphism in the developing gut microbiome of northern elephant seals. Mol. Ecol..

[18] Tian J, Du J, Han J, Song X, Lu Z (2020). Age-related differences in gut microbial community composition of captive spotted seals (*Phoca largha*). Mar. Mamm. Sci..

[19] Wu GD (2011). Linking long-term dietary patterns with gut microbial enterotypes. Science.

[20] Bigg MA, Ridgeway SH, Harrison RJ (1981). Harbour seal: *Phoca vitulina* and *P. largha*. Handbook of Marine Mammals.

[21] Parracho H, McCartney AL, Gibson GR (2007). Probiotics and prebiotics in infant nutrition. Proc. Nutr. Society.

[22] Marques TM (2010). Programming infant gut microbiota: Influence of dietary and environmental factors. Curr. Opin. Biotechnol..

[23] Palmer C, Bik EM, DiGiulio DB, Relman DA, Brown PO (2007). Development of the human infant intestinal microbiota. PLoS Biol..

[24] Mitsuoka T (1992). Intestinal flora and aging. Nutr. Rev..

[25] Bowen W, Oftedal O, Boness D (1992). Mass and energy transfer during lactation in a small phocid, the harbor seal (*Phoca vitulina*). Physiol. Zool..

[26] Bowen WD, Boness DJ, Iverson SJ (1999). Diving behaviour of lactating harbour seals and their pups during maternal foraging trips. Can. J. Zool..

[27] Jørgensen C, Lydersen C, Brix O, Kovacs KM (2001). Diving development in nursing harbour seal pups. J. Exp. Biol..

[28] Muelbert MMC, Bowen WD (1993). Duration of lactation and postweaning changes in mass and body composition of harbour seal, *Phoca vitulina*, pups. Can. J. Zool..

[29] Kim M, Cho H, Lee WY (2020). Distinct gut microbiotas between southern elephant seals and Weddell seals of Antarctica. J. Microbiol..

[30] Kershaw JL, Hall AJ (2016). Seasonal variation in harbour seal (*Phoca vitulina*) blubber cortisol—A novel indicator of physiological state?. Sci. Rep..

[31] Madison A, Kiecolt-Glaser JK (2019). Stress, depression, diet, and the gut microbiota: Human–bacteria interactions at the core of psychoneuroimmunology and nutrition. Curr. Opin. Behav. Sci..

[32] Thompson PM, Miller D, Cooper R, Hammond PS (1994). Changes in the distribution and activity of female harbour seals during the breeding season: implications for their lactation strategy and mating patterns. J. Anim. Ecol..

[33] Raulo A (2018). Social behaviour and gut microbiota in red-bellied lemurs (*Eulemur rubriventer*): In search of the role of immunity in the evolution of sociality. J. Anim. Ecol..

[34] Song SJ (2013). Cohabiting family members share microbiota with one another and with their dogs. Elife.

[35] Fernández-Martin EM, Heckel G, Schramm Y, García-Aguilar MC (2016). The timing of pupping and molting of the Pacific harbor seal, *Phoca vitulina richardii*, at Punta Banda Estuary, Baja California, Mexico. Cienc. Mar..

[36] Oates, S. C. Survival, movements, and diet of juvenile harbor seals along central California. [Master’s thesis, San Jose State University]. (2005). 10.31979/etd.ra96-xhge.

[37] Germain LR, Mccarthy MD, Koch PL, Harvey JT (2012). Stable carbon and nitrogen isotopes in multiple tissues of wild and captive harbor seals (*Phoca vitulina*) off the California coast. Mar. Mamm. Sci..

[38] Brassea-Pérez, E., Schramm, Y., Heckel, G., Chong-Robles, J. & Lago-Lestón, A. Metabarcoding analysis of the Pacific harbor seal diet in Mexico. *Mar. Biol. 166*, (2019). 10.1371/journal.pone.0221770 PMC6715212 31465508

[39] Davis TA, Nguyen HV, Costa DP, Reeds PJ (1995). Amino acid composition of pinniped milk. Comp. Biochem. Physiol. B Biochem. Mol. Biol..

[40] Sauvé, C. C., van de Walle, J., Hammill, M. O., Arnould, J. P. Y. & Beauplet, G. Stomach temperature records reveal nursing behaviour and transition to solid food consumption in an unweaned mammal, the harbour seal pup (*Phoca vitulina*). *PLoS ONE* **9**, (2014). 10.1371/journal.pone.0090329 PMC3936010 24587327

[41] Fernández Martín, E. M. Fenología de los nacimientos, estado de salud de las crías, y estructura genética poblacional de Phoca vitulina richardii en México [Doctoral thesis, Universidad Autónoma de Baja California, Mexico]. (2018).

[42] Gresse R (2017). Gut microbiota dysbiosis in postweaning piglets: Understanding the keys to health. Trends Microbiol..

[43] Sommer F (2016). The gut microbiota modulates energy metabolism in the hibernating brown bear *Ursus arctos*. Cell Rep..

[44] Ni Y (2021). Distinct composition and metabolic functions of human gut microbiota are associated with cachexia in lung cancer patients. ISME J..

[45] Pacífico C (2021). Unveiling the bovine epimural microbiota composition and putative function. Microorganisms.

[46] Fenn K (2017). Quinones are growth factors for the human gut microbiota. Microbiome.

[47] Rodríguez, J. M. *et al.* The composition of the gut microbiota throughout life, with an emphasis on early life. *Microb. Ecol. Health Disease* **26**, (2015). 10.3402/mehd.v26.26050 PMC4315782 25651996

[48] Thompson PM, Mackay A, Tollit DJ, Enderby S, Hammond PS (1998). The influence of body size and sex on the characteristics of harbour seal foraging trips. Can. J. Zool..

[49] van Parijs SM, Thompson PM, Tollit DJ, Mackay A (1997). Distribution and activity of male harbour seals during the mating season. Anim. Behav..

[50] Bjorkland RH (2015). Stable isotope mixing models elucidate sex and size effects on the diet of a generalist marine predator. Mar. Ecol. Prog. Ser..

[51] Schwarz D (2018). Large-scale molecular diet analysis in a generalist marine mammal reveals male preference for prey of conservation concern. Ecol. Evol..

[52] Boulva J (1975). Temporal variations in birth period and characteristics of newborn harbour seals. Rapports et procPs-verbaux, Reunions du Conseil International pour I’Exploration de la Mer.

[53] Bhute SS, Ghaskadbi SS, Shouche YS, Kalia VC, Shouche Y, Purohit HJ, Rahi P (2017). Rare biosphere in human gut: A less explored component of human gut microbiota and its association with human health. Mining of Microbial Wealth and MetaGenomics.

[54] Brown RF, Mate BR (1983). Abundance, movements, and feeding habits of harbor seals, *Phoca vitulina*, at Netarts and Tillamook Bays, Oregon. Fishery Bull..

[55] Higgins R (2000). Bacteria and fungi of marine mammals: A review. Can. Veterinary J..

[56] Gilbert MJ (2018). *Campylobacter blaseri* sp. nov., isolated from common seals (*Phoca vitulina*). Int. J. Syst. Evolut. Microbiol..

[57] Agnese ED (2020). Comparative microbial community analysis of fur seals and salmon aquaculture in Tasmania. Authorea..

[58] Rivas AJ, Lemos ML, Osorio CR (2013). *Photobacterium damselae* subsp. damselae, a bacterium pathogenic for marine animals and humans. Front. Microbiol..

[59] Fouz B, Toranzo AE, Milan M, Amaro C (2000). Evidence that water transmits the disease caused by the fish pathogen *Photobacterium damselae* subsp. damselae. J. Appl. Microbiol..

[60] Hundenborn, J., Thurig, S., Kommerell, M., Haag, H. & Nolte, O. Severe Wound Infection with *Photobacterium damselae* ssp. *damselae* and *Vibrio harveyi*, following a laceration injury in marine environment: A case report and review of the literature. *Case Rep. Med*. **2013**, (2013). 10.1155/2013/610632 PMC3792539 24171004

[61] Lubinsky-Jinich D, Schramm Y, Heckel G (2017). The Pacific Harbor Seal’s (*Phoca vitulina richardii*) breeding colonies in Mexico: Abundance and distribution. Aquat. Mamm..

[62] Arias-Del Razo A (2017). Distribution of four pinnipeds (*Zalophus californianus*, *Arctocephalus philippii townsendi*, *Phoca vitulina richardii*, and *Mirounga angustirostris*) on Islands off the west coast of the Baja California Peninsula, Mexico. Aquat. Mamm..

[63] Caporaso JG (2011). Global patterns of 16S rRNA diversity at a depth of millions of sequences per sample. Proc. Natl. Acad. Sci. USA..

[64] Kozich JJ, Westcott SL, Baxter NT, Highlander SK, Schloss PD (2013). Development of a dual-index sequencing strategy and curation pipeline for analyzing amplicon sequence data on the MiSeq Illumina sequencing platform. Appl. Environ. Microbiol..

[65] Robertson KM, Lauf ML, Morin PA (2018). Genetic sexing of pinnipeds: A real-time, single step qPCR technique. Conserv. Genet. Resour..

[66] Martin M (2011). Cutadapt removes adapter sequences from high-throughput sequencing reads. EMBnet.journal.

[67] Callahan BJ (2016). DADA2: High-resolution sample inference from Illumina amplicon data. Nat. Methods.

[68] R Core Team. R: A language and environment for statistical computing. *R Foundation for Statistical Computing, Vienna, Austria*. https://www.r-project.org/ (2019). Accessed 3 June 2021.

[69] McMurdie, P. J. & Holmes, S. Phyloseq: An R package for reproducible interactive analysis and graphics of microbiome census data. *PLoS ONE* **8**, (2013). 10.1371/journal.pone.0061217 PMC3632530 23630581

[70] Oksanen, J. *et al.* vegan: Community Ecology Package. R package version 2.5-4. https://cran.r-project.org/package=vegan (2019). Accessed 3 June 2021.

[71] Andersen KS, Kirkegaard RH, Karst SM, Albertsen M (2018). ampvis2: An R package to analyse and visualise 16S rRNA amplicon data. bioRxiv..

[72] Wickham H (2016). ggplot2: Elegant Graphics for Data Analysis.

[73] Salinas, H. & Ramirez-Delgado, D. ecolTest: Community Ecology Tests. (2021).

[74] Love MI, Huber W, Anders S (2014). Moderated estimation of fold change and dispersion for RNA-seq data with DESeq2. Genome Biol..

[75] Lozupone C, Knight R (2005). UniFrac: A new phylogenetic method for comparing microbial communities. Appl. Environ. Microbiol..

[76] Lozupone C, Lladser ME, Knights D, Stombaugh J, Knight R (2011). UniFrac: An effective distance metric for microbial community comparison. ISME J..

[77] Martinez Arbizu, P. pairwiseAdonis: Pairwise multilevel comparison using adonis. R package version 0.4. (2020).

[78] Douglas GM (2020). PICRUSt2 for prediction of metagenome functions. Nat. Biotechnol..

[79] Caspi R (2020). The MetaCyc database of metabolic pathways and enzymes-A 2019 update. Nucleic Acids Res..

