# Supplementary material for: Age as a primary driver of the gut microbial composition and function in wild harbor seals
Source: Sci Rep. 2022 Aug 27;12:14641. doi: 10.1038/s41598-022-18565-2 (PMC9420123; doi:10.1038/s41598-022-18565-2)
Supplement: Supplementary file 1 — Supplementary Information. [file 41598_2022_18565_MOESM1_ESM.pdf]

Supplementary information

Age as a primary driver of the gut microbial composition and function in wild harbor seals

Pacheco-Sandoval, A.[1], Lago-Lestón, A.[2], Abadía-Cardoso, A.[3], Solana-Arellano, E.[4], Schramm, Y.[3]*

[1] Posgrado de Ciencias de la Vida. Centro de Investigación Científica y de Educación Superior de Ensenada, Ensenada, Baja California, Mexico.

[2] Departamento de Innovación Biomédica. Centro de Investigación Científica y de Educación Superior de Ensenada, Ensenada, Baja California, Mexico.

[3] Universidad Autónoma de Baja California. Facultad de Ciencias Marinas, Ensenada, Baja California, Mexico.

[4] Departamento de Ecología Marina. Centro de Investigación Científica y de Educación Superior de Ensenada, Ensenada, Baja California, Mexico.

**Corresponding author: yschramm@uabc.edu.mx

Table of Contents

- **Supplementary Figure S1.** Microbial structure and diversity between fecal and rectal samples from two pups.

- **Supplementary Figure S2.** Sampling location.

- **Supplementary Figure S3.** Harbor seal's *phyla.*

- **Supplementary Figure S4.** Microbial profiles of harbor seals.

- **Supplementary Figure S5.** Beta diversity of variables with statistically insignificant outcomes.

- **Supplementary Table S1.** ASV count table with taxonomy.

- **Supplementary Table S2.** Core microbiome (ASVs) comparison between adults and pups.

- **Supplementary Table S3**. Adonis pairwise comparisons of microbial composition between age and sex interactions, calculated on UniFrac distance matrix.

- **Supplementary Table S4.** ASVs shared between female adults and pups and between male adults and pups.

Supplementary Figure S1- Microbial comparison between fecal and rectal samples of two harbor seal pups.

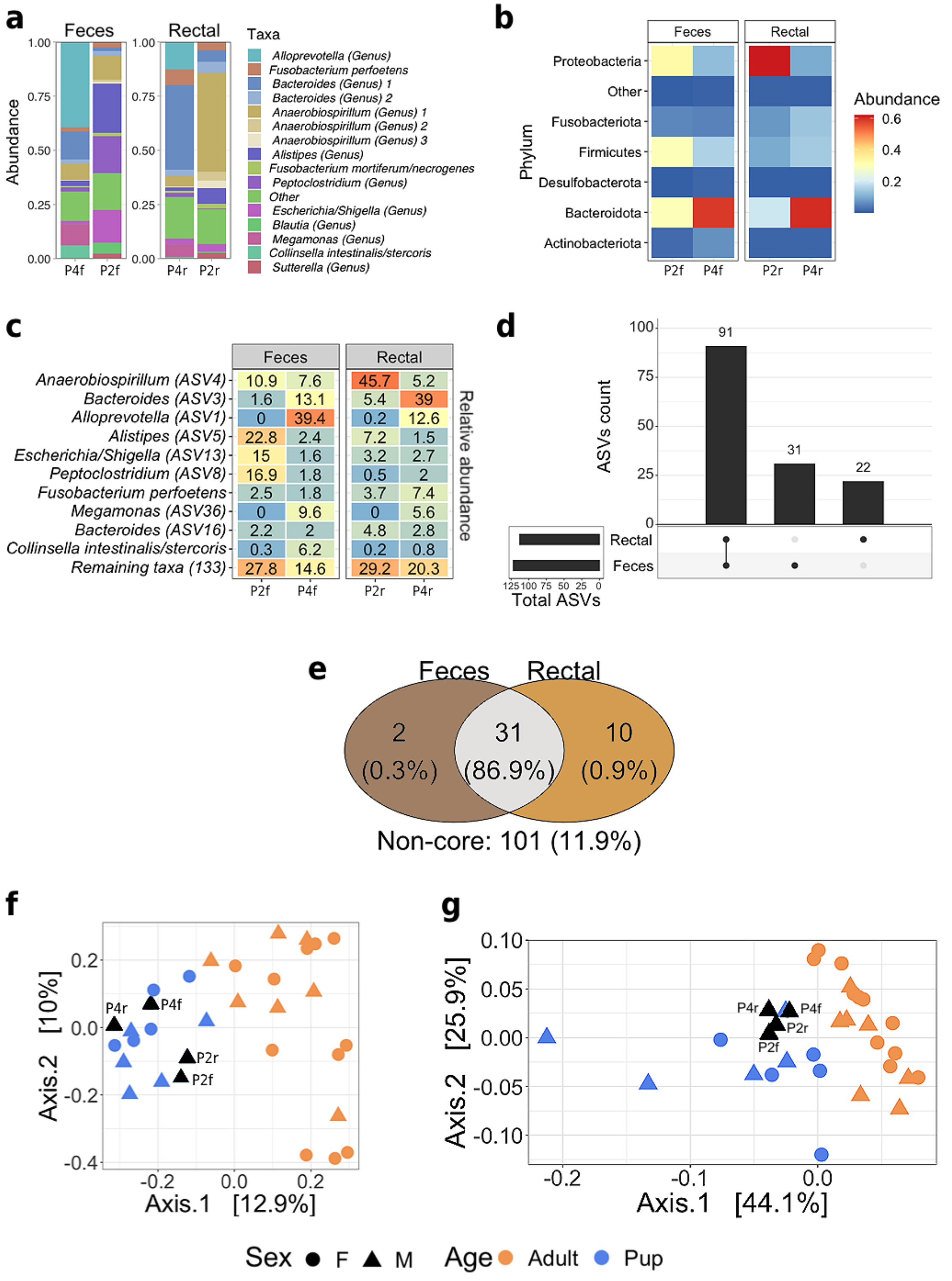

Supplementary Figure S1. Microbial structure and diversity between fecal and rectal samples from two pups. a) Microbial profiles of two harbor seal pups sorted by sample type (feces and rectal). For a user-friendly graphical representation**,** only the top 15 ASVs are shown; the rest are grouped under "Other". b) Phyla with a relative abundance> 1%. c) Top 10 most abundant ASV detected in the microbiome of harbor seals. The number of ASV is shown when the species is unassigned. d) Number of unique (single dot), shared (connected dots), and total (horizontal bars) ASVs in fecal and rectal samples. e) The core microbiome between fecal and rectal samples. The core group consisted of ASVs with an 80% prevalence and a relative abundance>= 0.01%. The Venn diagram shows the number of ASVs and their relative abundance in each core group. Principal Coordinate Analysis (PCoA) on unweighted (f) and weighted (g) UniFrac distances based on age groups. Feces and rectal samples from two pups are highlighted in black.

Supplementary Figure S2. Sampling location

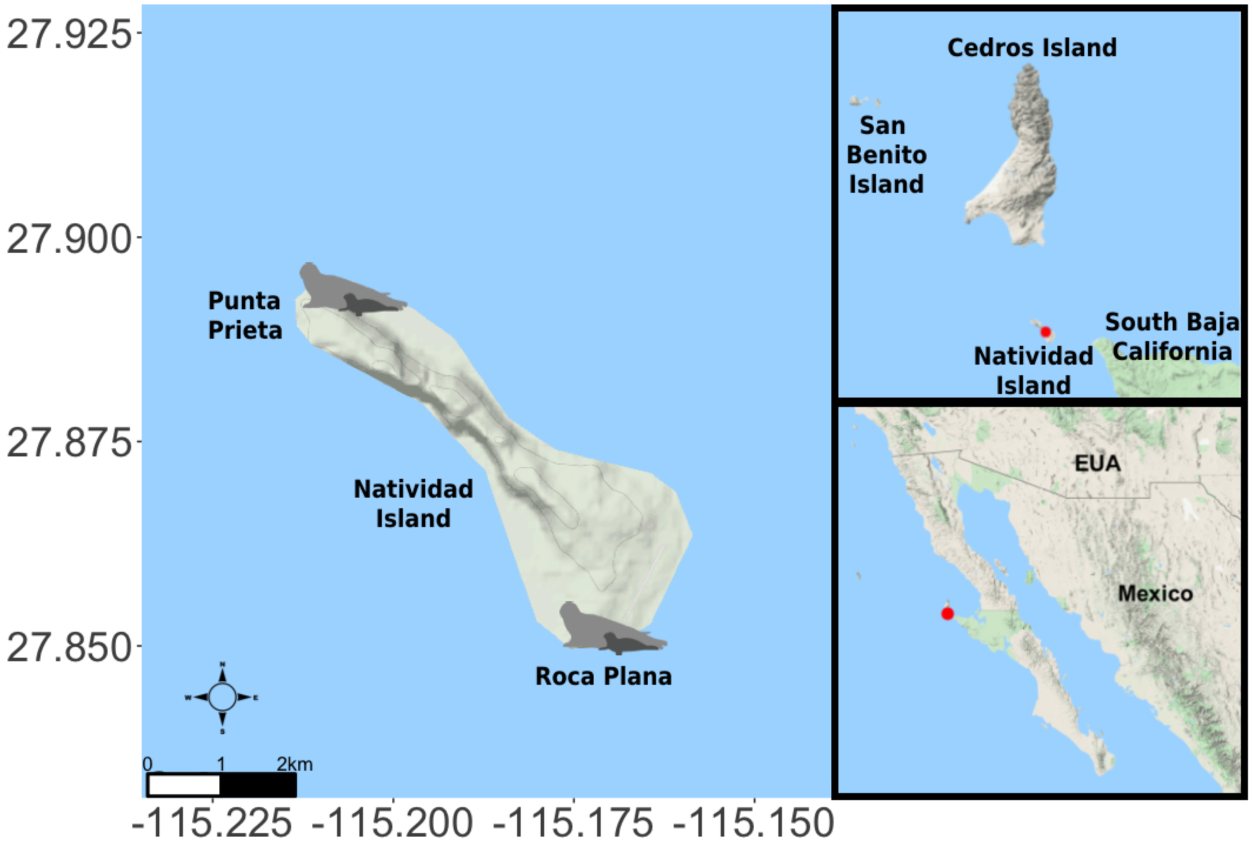

Supplementary Figure S2. Sampling locations of feces (adults) and rectal samples (pups) from harbor seals (*Phoca vitulina richardii*) at Natividad Island. The map was created in RStudio v.3.6.2 packages: *ggmap* v.3.0.0, (https://github.com/dkahle/ggmap), *ggplot2* v.3.3.5 (https://ggplot2.tidyverse.org), and *ggsn* v.0.5.0 (https://github.com/oswaldosantos/ggsn).

Supplementary Figure S3. Harbor seal's *phyla*

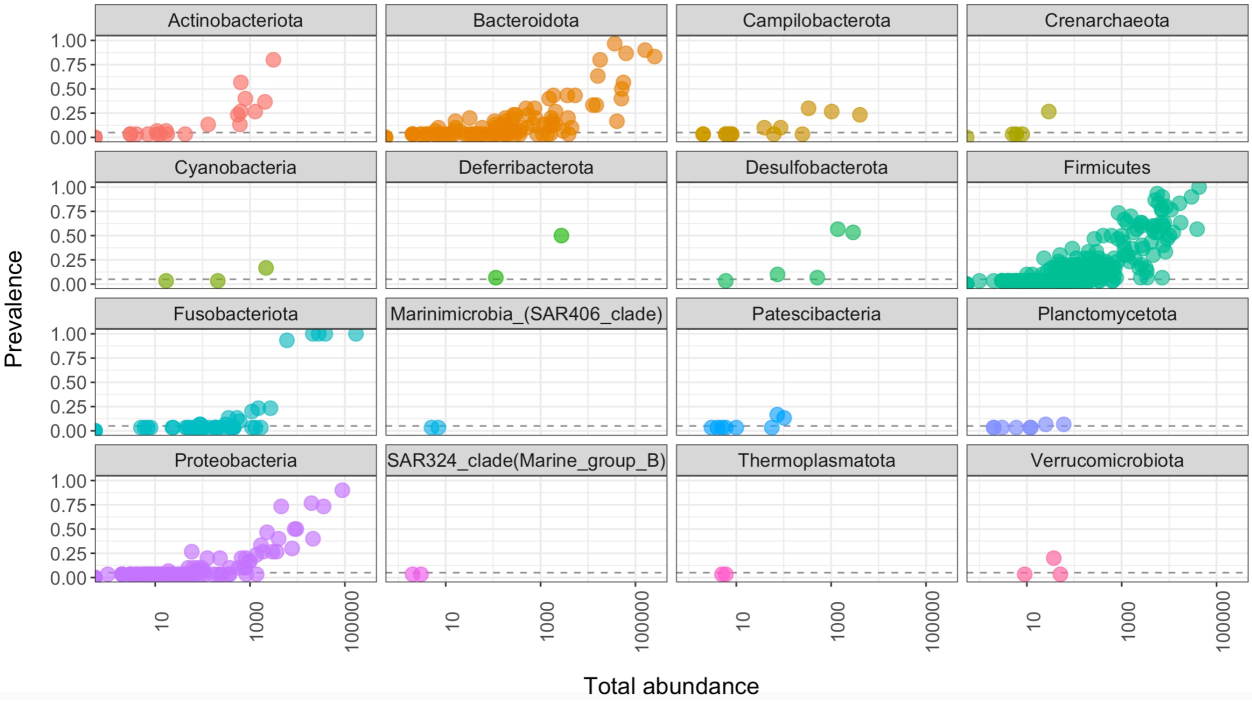

Supplementary Figure S3*. Phyla* detected in the harbor seal's pup and adult samples from Natividad Island. The dashed line denotes a prevalence threshold of 5%.

Supplementary Figure S4. Microbial profiles of harbor seals

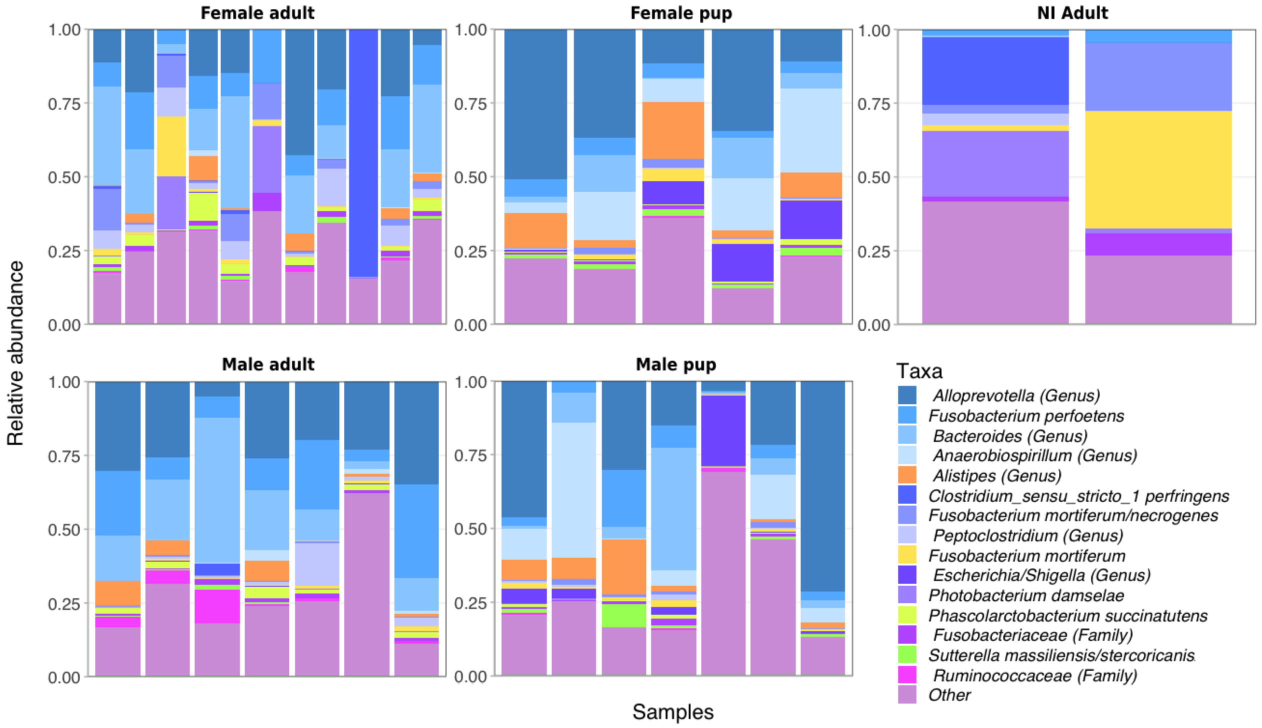

Supplementary Figure S4. Microbial profiles of 32 harbor seals sorted by age and sex categories. For a user-friendly graphical representation, only the top 15 ASVs are shown; the rest are grouped under "Other". NI= Non-identified sex.

Supplementary Table S3. **Adonis pairwise comparisons of microbial composition between age and sex interactions, calculated on UniFrac distance matrix.**

Distance metric	Comparison variables	Sums of Sqs	F. Model	R^2	*p*-value	*p*-adjusted
Unweighted UniFrac	Male pup vs. female pup	0.307	1.089	0.098	0.35	1
	Male adult vs. female adult	0.443	1.284	0.074	0.138	0.828
	Male pup vs. female adult	0.948	2.742	0.146	0.001	0.006*
	Male pup vs. male adult	0.764	2.481	0.171	0.001	0.006*
	Female pup vs. female adult	0.791	2.387	0.146	0.001	0.006*
	Female pup vs. male adult	0.620	2.213	0.181	0.004	0.024*
Weighted UniFrac	Male pup vs. female pup	0.064	1.690	0.145	0.114	0.684
	Male adult vs. female adult	0.037	1.957	0.109	0.129	0.774
	Male pup vs. female adult	0.274	9.854	0.381	0.001	0.006*
	Male pup vs. male adult	0.204	6.530	0.352	0.001	0.006*
	Female pup vs. female adult	0.159	7.314	0.343	0.001	0.006*
	Female pup vs. male adult	0.084	3.574	0.263	0.007	0.042*

*Significant diferences

Supplementary Figure S5- Beta diversity of variables with statistically insignificant outcomes.

Beta diversity between sexes

The variable "sex" in the linear mixed model showed homogeneity of dispersion across sex groups in both distance matrices (unweighted and weighted UniFrac). However, we did not observe significant differences in harbor seals' microbiota composition between females and males (Supplementary Fig. S5 a-b).

Beta diversity between the sexes of pups

When the number of microorganisms was not considered, individual variation in community structure was greater for male pups than for female pups (unweighted UniFrac, betadisper-test: p= 0.025). Individual dispersion was homogenous when the abundance of microorganisms was evaluated (betadisper-test: p= 0.665); however, differences in microbiota composition across pup sexes were not significant (Supplementary Fig. S5 c-d).

Beta diversity between the sexes of adults

Female and male adults showed dispersion homogeneity in both distance matrices (unweighted and weighted UniFrac). However, variations in microbiota composition between female and male adults were not significant (Supplementary Fig. S5 e-f).

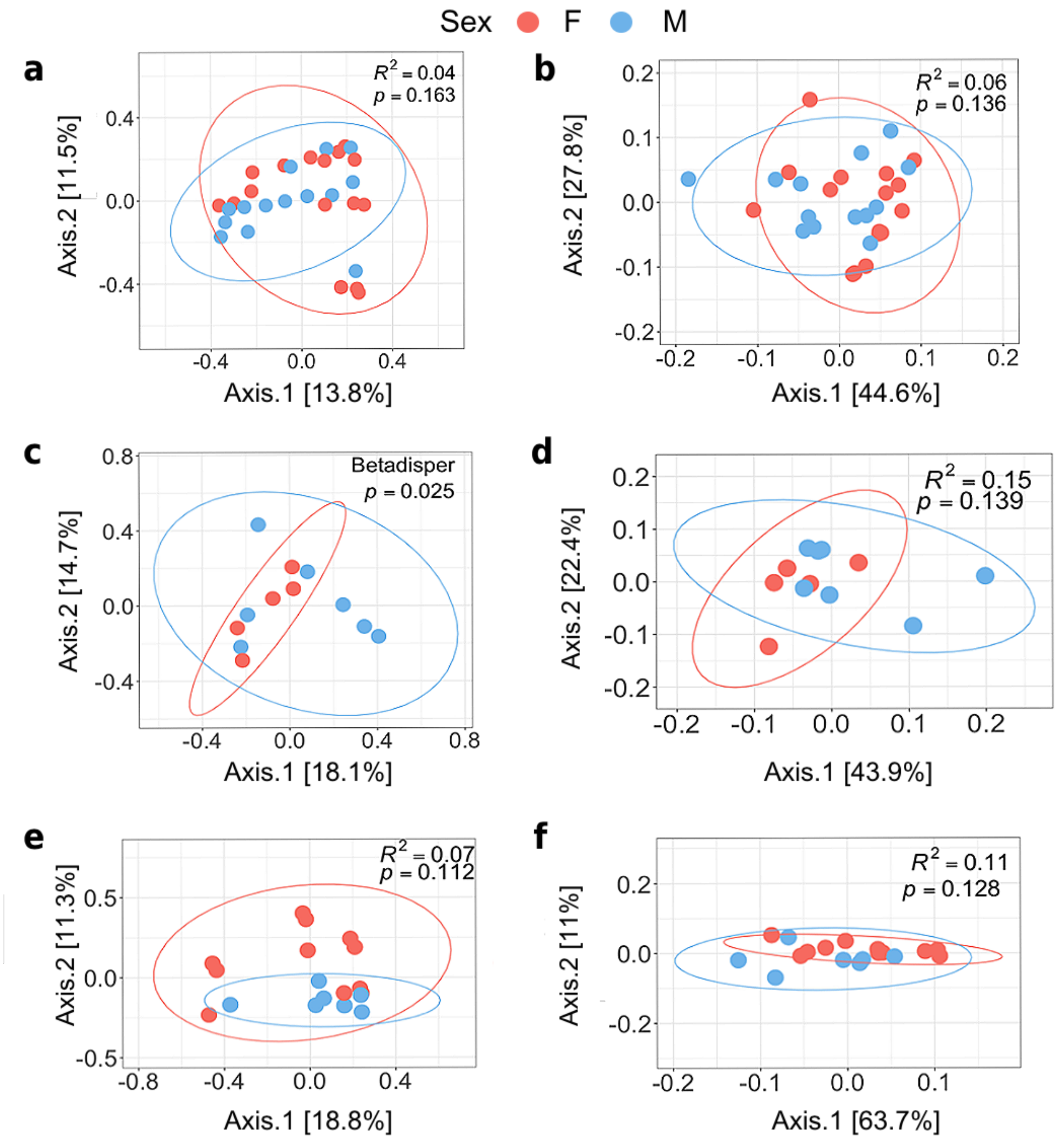

Supplementary Figure S5. No significant changes were observed in the microbial diversity between harbor seals' sexes (a-b), between female and male harbor seal pups (c-d), or between female and male harbor seal adults. Principal Coordinate Analysis (PCoA) on unweighted (a, c, e) and weighted (b, d, f) UniFrac distances matrices. Ellipses represent a 90% confidence interval. F= female, M= male
